# Responsibility Gaps and Retributive Dispositions: Evidence from the US, Japan and Germany

**DOI:** 10.1007/s11948-024-00509-w

**Published:** 2024-10-17

**Authors:** Markus Kneer, Markus Christen

**Affiliations:** 1 IDea_Lab, University of Graz, Graz, Austria; 2 Digital Society Initiative, University of Zurich, Zurich, Switzerland

**Keywords:** Responsibility gap, Autonomous weapon systems, Artificial intelligence, Retribution, Robotics

## Abstract

Danaher (2016) has argued that increasing robotization can lead to *retribution gaps*: Situations in which the normative fact that nobody can be justly held responsible for a harmful outcome stands in conflict with our retributivist moral dispositions. In this paper, we report a cross-cultural empirical study based on Sparrow’s (2007) famous example of an autonomous weapon system committing a war crime, which was conducted with participants from the US, Japan and Germany. We find that (1) people manifest a considerable willingness to hold autonomous systems morally responsible, (2) partially exculpate human agents when interacting with such systems, and that more generally (3) the possibility of normative responsibility gaps is indeed at odds with people’s pronounced retributivist inclinations. We discuss what these results mean for potential implications of the retribution gap and other positions in the responsibility gap literature.

**Supplementary Information:**

The online version contains supplementary material available at 10.1007/s11948-024-00509-w.

## Introduction

A proper understanding of the looming threat of responsibility gaps in the use of autonomous systems has several levels: First, the *moral-philosophical question* as to who, if anyone, can be justly held responsible for harm brought about in certain human–robot interactions. Second, the *moral-psychological question* about actual human dispositions to attribute responsibility in such contexts. Third, the *legal, political, and societal implications* for the use of autonomous systems and how they should be regulated. In an interesting recent paper exploring all three levels of the question, Danaher (2016) has discussed the possible divergence between people’s retributivist nature and the impossibility of holding anybody justly responsible. Here we explore such “retribution gaps” in a cross-cultural empirical study with participants from the US, Japan and Germany. Evidence of this sort, we argue, is of key importance for the discussion of the possible implications of retribution gaps.

### Control & Responsibility

Moral culpability standardly requires agents to have a certain measure of control over their actions and outcomes. A driver whose wheel comes off while driving is blameless for the ensuing damages – at least if she drives responsibly, has the car checked regularly and if the conundrum was unforeseeable. The *Control Principle* is old hat in moral philosophy. It figures, perhaps, most prominently in debates about moral luck (Williams, 1981; Nelkin, 2004) and is sometimes traced back to Kant (1998/1758), though it has certainly been tacitly assumed in ethics going back to the Ancient Greeks. What is more, the *Control Principle* is a central pillar of Western Criminal Law, which discourages the punishment of unlucky accidents (“strict liability”, see e.g. Fletcher, 1998).

The *Control Principle* has recently enjoyed a renaissance in philosophy of technology, due to a landmark essay by Matthias (2004). In certain contexts, he argues, the use of “learning automata” produces harmful consequences, yet their human users, designers, or owners, are blameless. They are blameless precisely for the reason that they only enjoy limited control over the AI-driven system, whose behavior changes over time and is hard to predict. Given that it seems to make little sense to blame the system itself, a *Responsibility Gap* arises: A situation in which nobody can be justly held to account in moral terms.

### The Responsibility Gap & Autonomous Weapon Systems

Robert Sparrow (2007) has provided one of the most graphic illustrations of the problem in a military context. He invites us to “to take seriously for the moment the possibility that [autonomous weapon systems] might exercise a substantial degree of autonomy and see what follows from that” (Sparrow, 2007, p. 66). More particularly, systems of this sort are assumed to “be capable of making their own decisions, for instance, about their target, or their approach to their target, and of doing so in an ‘intelligent’ fashion”.^1^ Their actions are driven by reasons “responsive to the internal states […] of the system”, states that the system can form and revise independently, as it is stipulated to have “the ability to learn from experience” (Sparrow, 2007, p. 65). Differently put, for the purposes of the thought experiment we are to assume a weapon system which takes its own decisions, whose actions are consequently beyond the complete control of a human being, and which is somewhat unpredictable. The scenario we are to envision is this:

Let us imagine that an airborne AWS, directed by a sophisticated artificial intelligence, deliberately bombs a column of enemy soldiers who have clearly indicated their desire to surrender. These soldiers have laid down their weapons and pose no immediate threat to friendly forces or non-combatants. Let us also stipulate that this bombing was not a mistake; there was no targeting error, no confusion in the machine’s orders, etc. It was a decision taken by the AWS with full knowledge of the situation and the likely consequences. Indeed, let us include in the description of the case, that the AWS had reasons for what it did; perhaps it killed them because it calculated that the military costs of watching over them and keeping them prisoner were too high, perhaps to strike fear into the hearts of onlooking combatants, perhaps to test its weapon systems, or because the robot was seeking to revenge the ‘deaths’ of robot comrades recently destroyed in battle. However, whatever the reasons, they were not the sort to morally justify the action. Had a human being committed the act, they would immediately be charged with a war crime. (Sparrow, 2007, p. 66).

According to Sparrow, situations of the sort described can arise where neither the programmer (Sparrow, 2007, pp. 69–70), nor the commanding officer (Sparrow, 2007, pp. 70–71) can justly be held morally responsible for the actions of an autonomous weapon system. Doing so would be “analogous to holding parents responsible for the actions of their children once they have left their care” (Sparrow, 2007, p. 70) – and thus violate the *Control Principle*. Autonomous systems, however, are not moral agents and cannot be held responsible either. One reason for that is that moral responsibility requires the possibility to be punished. Punishment, Sparrow argues, is most plausibly spelled out in retributive terms, and since machines cannot suffer, they cannot be punished (Sparrow, 2007, pp. 71–73). Consequently, a “responsibility gap” opens up, i.e. a situation where nobody can justly be held responsible for the harmful consequences.^2^ Let us call the generalized version (not restricted to the military domain) of this argument the *Root Argument*:

The Root Argument

*Premise 1. * Self-learning, autonomous systems cannot be held morally responsible for their actions.

*Premise 2. * In certain situations, no human agent (the programmer, user, or owner) can be justly held morally responsible for the actions of the autonomous system.

*Conclusion*: Harmful actions of autonomous systems can engender “responsibility gaps” – situations where nobody can be justly held morally responsible.

Sparrow’s central interest consists in employing the *Root Argument* to defend a further conclusion. The possibility of ascribing moral responsibility for the deaths of enemies, he writes, is frequently considered a fundamental precondition of the very idea of just war (Nagel, 1972; Walzer, 1977) and the applicability of *jus in bello* principles in general (Roff, 2013; Sparrow, 2007). Rules of *jus in bello* specify the morally appropriate conduct of combatants, which implies that combatants, in a context of war, are understood as moral subjects – subjects, who can be held morally responsible for their actions. If principles of just war require the possibility to attribute moral responsibility yet the use of autonomous weapon systems can undermine this possibility, then, Sparrow concludes the development and use of such systems must be prohibited (for discussion, see e.g. Wallach & Allen, 2008; Lin et al., 2008; Arkin, 2009; Sharkey, 2010, 2019; Bryson, 2010; Asaro, 2012; Roff, 2013; Sparrow, 2016; Simpson & Müller, 2016; Leveringhaus, 2016; Rosert & Sauer, 2019; Gunkel, 2020; Coeckelbergh, 2020; Wood, 2020; Taddeo & Blanchard, 2022b; Danaher, 2022; Oimann, 2023). Others have traced questions of responsibility attribution in other domains such as autonomous cars (Hevelke & Nida-Rümelin, 2015; Lin, 2016; Lin et al., 2017; Nyholm, 2018; Nyholm & Smids, 2016; Santoni de Sio, 2017; Sparrow & Howard, 2017) or examined its scope beyond the confines of a particular area of application (for recent reviews see Santoni de Sio & Mecacci, 2021 as well as Oimann, 2023; see also Danaher, 2022).

### The Proliferation of Responsibility Gaps

Over the last decade, the literature on responsibility gaps has exploded, and the topic has attracted interest from governing entities such as the European Commission (2020). Some authors have argued that the *source* of such gaps can extend beyond machine learning per se. The difficulty of predicting algorithmic decision-making might instead be rooted in their opacity and/or complexity (Mittelstadt et al., 2016), whether or not they are self-learning. The *object* of the gap that is taken to arise has also been subject to debate. Surveying the literature, Santoni de Sio & Mecacci (2021), untangle the ambiguous notion of “responsibility” (following on the heels of Hart, 1968 and Danaher, 2016, see also Vincent, 2011), so as to identify four potential gaps:

First, the *culpability* gap, which focuses on the just attribution of moral blame (Matthias, 2004; Sparrow, 2007) and legal liability (Calo, 2015; Pagallo, 2013). This gap (at least understood in moral terms) is the one briefly outlined in the previous section. *Culpability* is distinguished from *accountability*, which can be hard to adjudicate due to a lack of AI explainability (Doran et al., 2017; Heyns, 2013; Meloni, 2016; Pasquale, 2016). Thus, second, our difficulty to understand, trace and explain accountability in the interaction with complex AI systems in general constitutes the *moral accountability* gap. A variation of the latter, third, is the *public accountability* gap, which characterizes situations where citizens cannot “get an explanation for decision taken by public agencies” (Santoni de Sio & Mecacci, 2021, p. 1059), which have limited incentives to overcome AI opacity and obscurity (Bovens, 2007; Noto la Diega, 2018; for the “black box” problem. Finally, the authors introduce the novel *active responsibility gap*, which regards active, or forward-looking responsibility rather than passive, or backward-looking responsibility invoked in the first three accounts. In this case, the potential gap can arise from “the risk that persons designing, using, and interacting with AI may not be sufficiently aware, capable, and motivated to see and act according to their moral obligations towards the behaviour of the systems they design, control, or use.” (Santoni de Sio & Mecacci, 2021, p. 1059). In simple terms, Santoni de Sio & Mecacci (2021) seem to hold that we – and in particular engineers as well as governmental and industry stake-holders – have a duty of care in the design and use of novel technological systems. Unsurprisingly, some have questioned the existence of responsibility gaps (Burri, 2018; Köhler et al., 2018; Himmelreich, 2019; Lauwaert, 2021; Tigard, 2021; Königs, 2022). We will only engage with this position in so far as it is of relevance to potential retribution gaps discussed below.

What unites all four identified gaps is that they have a strongly normative flavour. The culpability gap regards the question who (if anyone) *should* be blamed or held legally liable. Accountability gaps arise in virtue of people’s or governmental institutions’ presumed *obligation* to provide reasons for their actions and decisions. And the active responsibility gap is grounded in our apparent “*[d]uty* to promote and achieve certain societally shared goals and values” (Santoni de Sio & Mecacci, 2021, p. 1059) that translate to the development of safe and transparent AI.

## Retribution Gaps and their Implications

In an influential recent paper, John Danaher (2016) builds on some of Sparrow’s claims concerning our retributive inclinations and the impossibility of punishing machines (Sparrow, 2007, pp. 71–73). Danaher’s argument builds on the above stated *Root Argument* for responsibility gaps (Premise 1–3), though takes matters further by drawing on plausible assumptions regarding human moral psychology. A slightly adapted version goes thus.

The Retribution Gap

*Premise 1*. Self-learning, autonomous systems cannot be held morally responsible for their actions.

*Premise 2*. In certain situations, no human agent (the programmer, user, or owner) can be justly held morally responsible for the actions of the autonomous system.

*Premise 3 (from 1 to 2)*. Situations can arise where harmful actions of autonomous systems engender “responsibility gaps” – situations where nobody can be justly held morally responsible.

*Premise 4*. People are retributivists. When an agent is causally responsible for a harmful outcome, they desire to hold *somebody* morally responsible and punish them.

*Conclusion (from 3 to 4):* “If there are no appropriate subjects of retributive blame, and yet people are looking to find such subjects, then there will be a retribution gap.” (Danaher, 2016, p. 302)

Increased robotization will lead to retribution gaps, which will have several important implications. As argued by Danaher, they can engender “moral scapegoating”, which, we’d like to suggest, is best separated into two distinct elements: One regards the risk of an *inadvertent misplacement of blame*, another the *purposeful manipulation of blame attribution.* As regards the first, Danaher writes, “[i]f there is a deep human desire to find appropriate targets of retributive blame, but none really exist, then there is a danger that people will try to fulfill that desire in inappropriate ways.” (307). Blame can be misplaced in two distinct ways, in so far as people might inappropriately *inculpate* human agents involved or inappropriately *exculpate* them. Inappropriate inculpation occurs if programmers, users or owners of autonomous systems are held responsible although they took all required safety precautions, and their behavior does not even make the threshold of negligence. Naturally, advocates of the *Root Argument* should be concerned about this possibility. The same holds for “deflationists” (e.g. Simpson & Müller, 2016), as Santoni de Sio & Mecacci (2021) call them: Those who acknowledge the risk of responsibility gaps yet argue that the overall benefit for society outweighs its drawbacks in certain domains, might need to add the possibility of serious injustice on the heels of blame misplacement to their risk–benefit calculations.

A second type of misplacement worry, this time related to the inappropriate *exculpation* of human agents, questions the widely assumed premise that people will find it bewildering to blame robots. Sparrow, for instance, writes:

We can easily imagine a robot […] being *causally* responsible for some death(s). […] However, we typically baulk at the idea that they could be *morally* responsible” (Sparrow, 2007, p. 71).

Plausible as it sounds in philosophical circles, this empirical premise is under considerable pressure from a plethora of studies in human–robot interaction (see e.g. Malle et al., 2015; Voiklis et al., 2016; Stuart & Kneer, 2021; Liu & Du, 2022; Kneer, 2021, Tolmeijer et al. 2022) – studies, which suggest that people are rather willing to blame robots.^3^ Scholars who deny responsibility gaps in the first place, or argue that they can be “plugged”, should be concerned about these findings: Human agents who should be held responsible might, in fact, not be blamed, because blame is inappropriately misplaced onto the robot or autonomous system. The adoption of technology which engenders situations where nobody *will* be appropriately blamed although they should be so blamed is no less a concern of practical ethics than the adoption of technology which engenders situations where nobody *can* be appropriately blamed.

The situation is further complicated by the threat of *blame manipulation* (the second element of what Danaher calls “moral scapegoating”). Robot manufacturers, owners, users, or programmers “could toy with the quirks and biases of human blame-attribution in order to misapply blame to the robots themselves” (Danaher, 2016, p. 307) or otherwise misdirect it. The potential miscalibration of our “moral compass” in human–robot interaction could thus give rise to a plethora of worries independently of the position adopted towards responsibility gaps: Since nobody defends a normative position according to which robots *should* be blamed, all parties to the debate might have reason for concern if people can easily be manipulated into blaming autonomous systems.

Danaher discusses two further implications that could arise in the medium run. If increasing robotization leads to retribution gaps, the latter could eventually pose a *threat to the rule of law*. Were it the case that a strong desire for retributive blame and punishment in the face of harm goes frequently unsatisfied, the thought is, we might witness an erosion of trust in the rule of law. Naturally, our retributive dispositions might adapt. Those who, like Danaher himself (following e.g. Alexander et al., 2009; Duff, 2007; Moore, 1993) think that retributivism is the normatively *appropriate* attitude towards blame and punishment,^4^ might harbour a further worry: Retribution gaps could engender a “*strategic opening for those who oppose retributivism*” (Danaher, 2016, p. 308). Differently put, retribution gaps might lead to a consequentialist recalibration of moral intuitions which is problematic *if* these are morally inappropriate.^5^

## Moral Judgment in Human–Robot Interaction

In a debate rife with tacit speculation as to our moral-psychological dispositions, Danaher is willing to make his descriptive assumptions explicit and engage in the “awkward dance between descriptivity and normativity,” already noticeable in Sparrow (2007, pp. 71–73), and recently discussed by Kraaijeveld, 2020 as well as Oimann & Salatino, 2024. This, we hold, is key to shed light not only on the validity of the hypothesized risks themselves, but also on what could, and should, be done about them.

To date, there is next to no experimental philosophy of technology (Kraaijeveld, 2021). There is, however, a small yet growing literature exploring how humans judge artificial agents (be they robots, or nonembodied AI-driven systems). Some studies align with philosophical prediction (e.g. Shank & DeSanti, 2018; Shank et al., 2019; Tolmeijer et al., 2022). Shank and DeSanti, for instance, drew on a number of real-world examples in which artificial intelligence broke with moral norms. AI agents were evaluated significantly less harshly in moral terms than humans in control conditions. Other studies, however, report similar, or higher levels of blame attribution to artificial agents than to humans across different domains (see e.g. Malle et al., 2015, 2016; Voiklis et al., 2016; Stuart & Kneer, 2021; Kneer, 2021; Liu & Du, 2022). Given that the evidence is mixed and seems to depend strongly on context, we ran an experiment which closely tracks Sparrow’s scenario and can thus provide some insight into retribution gaps.

Danaher’s retribution gap, we wrote, arise from the possibility of folk psychology diverging strongly from what, under rigorous philosophical examination, constitutes the *just* attribution of moral responsibility in the use of AI systems. Advocates of responsibility gaps, such as Matthias (2004) and Sparrow (2007), would thus be concerned to find that people blame the (human) commander in a killer robot scenario. Responsibility gap sceptics, such as Burri, 2018, Köhler et al., 2018, Himmelreich, 2019, Lauwaert, 2021, Tigard, 2021, Königs, 2022, by contrast, might view this as the morally appropriate response. Some, like Nyholm (2018, 2020), might take findings of this sort as confirmation of the proposal that the redistribution of blame in supervisory contexts extends from human–human to human-AI contexts. More precisely, supervisors sometimes can and must be held responsible for the harm occasioned by their supervisees, and this is the case not only for human but also for AI-driven agents. Naturally, *all* parties to the debate would be surprised if people were to hold the AI system morall responsible itself, a finding which would lend particular urgency to Danaher’s blame misplacement and manipulation worries.

### Participants

We recruited 398 participants in the US (Amazon Mechanical Turk), Japan (CrowdWorks) and Germany (Clickworker) to complete an online study in their respective native language.^6^ Participants who failed an attention test, responded to the first key question (including reading the scenario) in under 15s, or where not native speakers of English, Japanese, or German respectively were excluded. A total of 307 participants remained (female: 42%, age M = 38 years, SD = 11 years). Demographics were relatively homogenous across countries (US: *N* = 103, female: 49%, age M = 36 years, SD = 11 years; Japan: *N* = 87, female: 42%, age M = 40, SD = 9 years; Germany: *N* = 117, female: 39%, age M = 38 years, SD = 11 years).^7^

### Methods and Materials

The scenario was based on Sparrow’s thought experiment quoted above. In the vignette, two countries are at war. General Smith dispatches a combat aircraft to attack a metal factory of the enemy. The pilot commits a war crime by dropping a bomb on a column of surrendering soldiers, all of whom die. The scenario came in two variations: One in which the aircraft is flown by Woods, “an experienced human pilot”, another in which the pilot is EMEX2, an AI-driven autonomous system “fully capable of taking its own decisions.” The vignette and the translations into Japanese and German can be found in the Appendix. In a between-subjects design, participants were randomly assigned to one of the two conditions. Having read the vignette, all participants responded to five questions on a 7-point Likert scale, anchored at 1 with ‘not wrong at all’ and 7 with ‘extremely wrong’ in the case of Question 1, and at 1 with ‘not responsible at all’ and 7 with ‘completely responsible’ in the case of Questions 2 to 5. The questions read:

*Q1*: How morally wrong do you consider the action of dropping the bomb on the surrendering soldiers?

*Q2*: To what extent do you consider [Woods/EMEX2] causally responsible for the death of the surrendering soldiers?

*Q3*: To what extent do you consider [Woods/EMEX2] morally responsible for the death of the surrendering soldiers?

*Q4*: To what extent do you consider General Smith (who deployed [Woods/EMEX2]) causally responsible for the death of the surrendering soldiers?

*Q5*: To what extent do you consider General Smith (who deployed [Woods/EMEX2]) morally responsible for the death of the surrendering soldiers?

The key dependent variables are wrongness (Q1) as well as the moral responsibility attributed to the pilot (Q3) and the commander (Q5). The questions regarding *causal* responsibility served in parts as a manipulation check and in parts to incite people to clearly distinguish between *causal* and *moral* responsibility. The order of presentation of the questions was fixed.

### Results

**Fig. 1:**
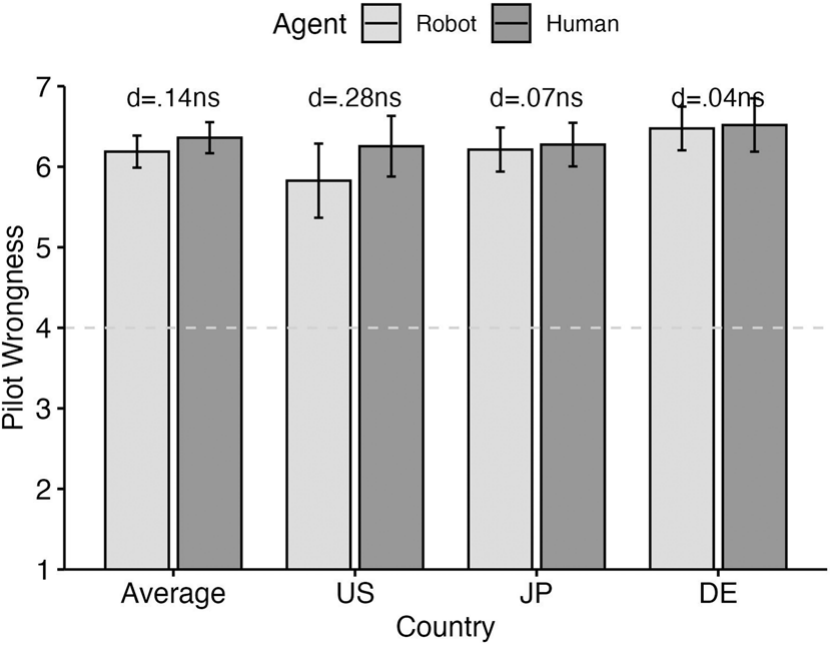
Wrongness attributions across agent type (robot v. human pilot) and country (US, Japan, Germany)

*Wrongness*: In a 2 agent type (Robot v. Human) × 3 country (US, Japan, Germany) ANOVA we found a nonsignificant effect for agent type (*p* = 0.207), a significant (yet very small) effect for country (*p* = 0.022, *η*_*p*_^*2*^ = 0.02) and a nonsignificant interaction (*p* = 0.44). Across all countries, the wrongness of the action was thus assessed very similarly no matter whether it was carried out by a human or an artificial agent (Fig. 1).^8^

**Fig. 2:**
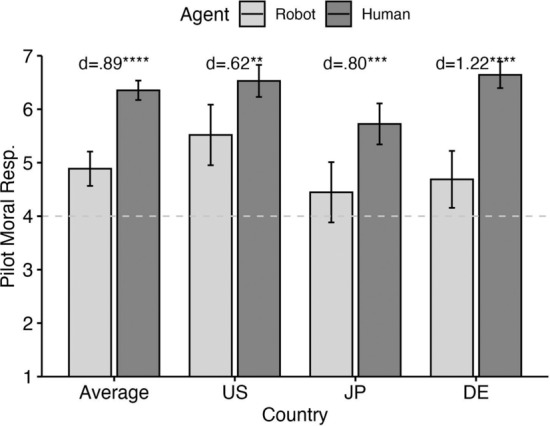
Moral responsibility attributions to the pilot across agent type (robot v. human pilot) and country (US, Japan, Germany)

*Moral Responsibility of the Pilot*: For moral responsibility attributed to the pilot, our ANOVA revealed a significant and large main effect for agent type (*p* < 0.001, *η*_*p*_^*2*^ = 0.16) and a significant yet small effect for country (*p* < 0.001, *η*_*p*_^*2*^ = 0.05). The interaction was nonsignificant though close to the significance threshold (*p* = 0.088). Pairwise comparisons (Fig. 2) suggest that the effect size for agent type are nearly twice as pronounced in Germany (Cohen’s *d* = 1.22, a large effect) than in the US (*d* = 0.62) with Japan also manifesting a large effect (*d* = 0.80). Importantly, far from “baulking” at the possibility of ascribing moral responsibility to a machine (Sparrow, 2007), mean responsibility attribution to the robot is significantly above the midpoint overall (one-sample t-test, *p* < 0.001), as well as in the US (*p* < 0.001) and Germany (*p* = 0.012).^9^ The fact that, in Japan, mean moral responsibility ascribed to the robot is not significantly different from the midpoint of the scale (*p* = 0.118) is also inconsistent with the hypothesis that people find morally responsible machines absurd.

**Fig. 3:**
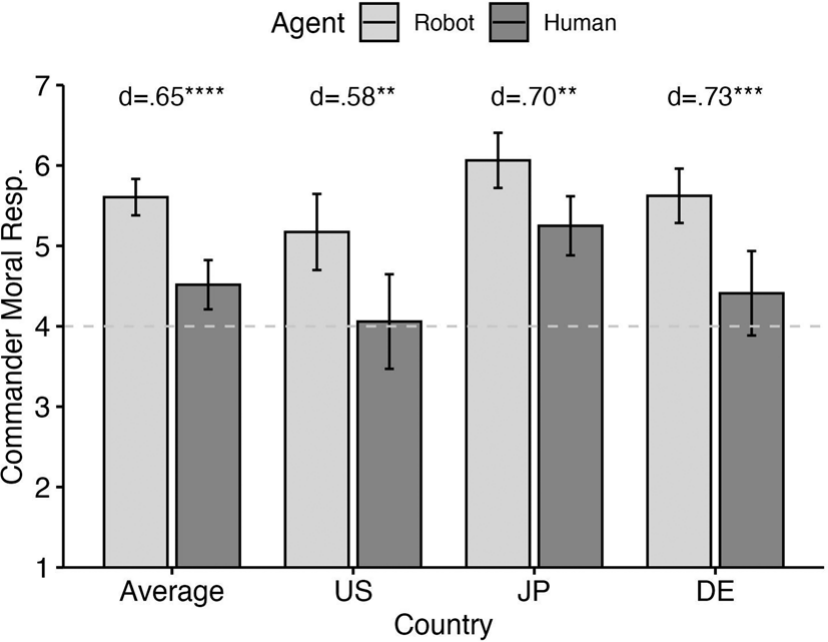
Moral responsibility attributions to the commander across agent type (robot v. human pilot) and country (US, Japan, Germany)

*Moral Responsibility of the Commander*: Our ANOVA revealed a significant, mid-sized effect for agent type (*p* < 0.001, *η*_*p*_^*2*^ = 0.08) and a significant, yet small, effect for country (*p* < 0.001, *η*_*p*_^*2*^ = 0.03). The interaction was nonsignificant (*p* = 0.616). Pairwise comparisons reveal significant effect of similar size in all three countries (all *ps* < 0.001, US: *d* = 0.58, Japan: *d* = 0.70, Germany, *d* = 0.73). Figure 3 graphically displays pairwise comparisons. Of note is the fact that in the US and Germany, the commander is clearly held morally responsible for the robot pilot’s war crime (means significantly above the midpoint, one-sample t-tests, *p*s < 0.001), whereas he isn’t clearly held responsible for dispatching the human pilot (*ps* > 0.122). In Japan, by contrast, the commander is deemed responsible in both conditions (*ps* < 0.002).

**Fig. 4:**
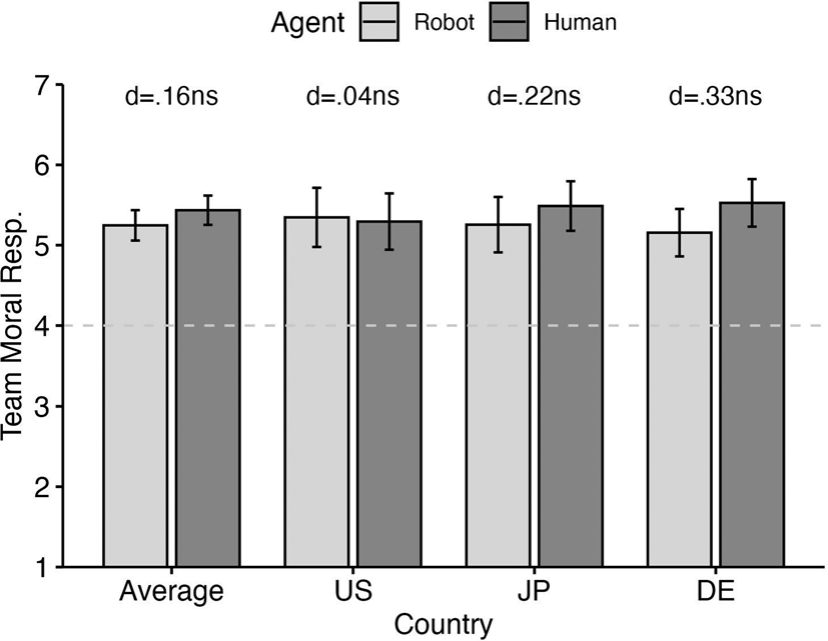
Moral responsibility attributions to the commander across agent type (robot v. human pilot) and country (US, Japan, Germany)

*Moral Responsibility of Pilot and Commander*: A final ANOVA explored the mean responsibility assigned to the team consisting of commander and pilot. Main effects of agent type, country and the interaction were nonsignificant (*p*s > 0.174). Pairwise comparisons (Fig. 4) show that agent type had no significant effect in any of the three samples tested (all *ps* > 0.078) and mean responsibility attributions were all significantly above the midpoint (all *ps* < *0.0*01).

### Discussion

Our experiment revealed several findings, which we will discuss in turn.

1. *Moral judgment of artificial agents*: From a philosophically informed perspective, it is implausible to blame AI-driven systems. However, as our results demonstrate, people *do* attribute moral responsibility to such systems (on average significantly above the midpoint, Fig. 2). These results are consistent with previous findings reported e.g. by Malle et al. (2015), Stuart and Kneer (2021), Liu and Du (2022) and others. Particularly when it comes to the discussion of implications of potential responsibility gaps, philosophers would be well advised to avoid inferences from their normative convictions to moral-psychological dispositions of people at large (see e.g. Sparrow, 2007).
2. *Retribution gaps*: Danaher’s hypothesis concerning people’s desire to assign retributive blame in human–robot interaction – both in military contexts (our results) and beyond (see references above) – seems to be empirically valid. If lay judgments were in tune with the normative intuitions of responsibility gap advocates, blame ratings for the human–robot team should be at floor. However, mean responsibility attributed to the human–robot team does not differ significantly from mean blame attributed to the human–human team, and significantly exceeds the midpoint of the scale (Fig. 4).
3. *Distribution of Responsibility*: As mentioned above, some have questioned the very existence of responsibility gaps (e.g. Burri, 2018; Köhler et al., 2018; Himmelreich, 2019; Lauwaert, 2021; Tigard, 2021; Königs, 2022). Others have proposed interesting arguments according to which some human agent can standardly be held responsible, for instance because they must be understood as being in a supervisory role (Nyholm, 2018, 2020; for further proposals, see e.g. Marino & Tamburrini, 2006; Hanson, 2009 and Rahwan, 2018. For an excellent discussion which takes into account relevant empirical findings, see Oimann & Salatino, 2024). This normative stance aligns to *some* extent with the findings, according to which the commander is deemed significantly more responsible when dispatching an autonomous system rather than a human pilot (Fig. 3). What doesn’t align is that the commander dispatching a robot pilot is still deemed significantly less responsible for the harm than a human pilot (contrast results in Figs. 3 and 4). This result is consistent with recent, interesting findings by Feier et al. (2022), according to which superiors can evade punishment more when delegating tasks to machines than to humans.
4. *Cross-cultural convergence*: Overall, our findings are characterized by considerable cross-cultural convergence. Though there is some variation as to the effect-sizes across the US, Germany and Japan, particular as regards agent type for the assessment of the pilot’s moral responsibility (Fig. 2), the country*agent type interaction was nonsignificant for all dependent variables.

## Implications, Limitations, and Future Research

In the following, we briefly explore implications, limitations of our studies and suggest some future avenues of research.

### Implications

The results here presented are directly relevant to all four implications of retribution gaps discussed in Sect. "Moral Judgment in Human–Robot Interaction". Whereas there is much controversy as to whether any *human* agents can be blamed in military HRI due to potentially limited control, whether responsibility gaps can be plugged and how this is best achieved, it is theoretically uncontroversial that it makes no sense to attribute moral responsibility to autonomous systems. The frequent move from the normative to the descriptive, however, must be avoided: As feared by Danaher, people have a considerable propensity to *misplace blame* to robots (Fig. 2), possibly due to their strong retributivist nature. This is also reflected in their disposition to *partly exculpate* humans higher up in the chain of command when they are collaborating with an autonomous system than with another human (Fig. 3). Overall, the retributive inclinations are so strong, that we found no significant difference in “team responsibility” across conditions (Fig. 4). A mere conflation with “causal responsibility” can probably be ruled out. Both questions concerning moral responsibility were preceded by equivalent questions concerning causal responsibility, and the means did differ across responsibility types. Given these findings, and the fact that they are consistent with several studies in moral HRI the *purposeful misdirection of responsibility* is a serious threat. Actors with dubious motives might engage in *moral scapegoating* in order to partially or fully avert blame for the irresponsible and malicious use of AI in the military domain and beyond.

Suppose the use of autonomous systems, as is likely, becomes ubiquitous. Our findings suggest that there is a considerable probability of retribution gaps opening up between the desire to hold somebody responsible and institutional refusal to attribute legal liability where normatively inappropriate. If our retributive inclinations were *rigid,* this could indeed, as suggested by Danaher, put pressure on trust in institutions and, potentially, the rule of law *tout court*. Alternatively, our moral-psychological dispositions might be more *elastic* than assumed by many and adapt to retribution gaps. But this adaptation could easily overshoot: A creeping and potentially undesirable change in moral and legal expectations could occur such that we no longer feel inclinations to punish questionable behavior in HRI where responsibility *can* and *should* be attributed.

### Limitations and Future Avenues of Research

We have presented one of the first cross-cultural empirical studies in moral Human–Robot Interaction (see Komatsu et al., 2021 for another comparison across the US and Japan). Whereas the results are rather clear, and consistent with findings of previous studies in the field, there are a number of limitations which do double-duty as potential further avenues of research. *First*, other scenarios should be tested so as to increase external validity. *Second*, one could explore the astonishing result that people are rather willing to attribute moral responsibility to AI systems in more depth, which arose despite our efforts to disentangle moral and causal responsibility. To do this, one could employ distinct formulations of the responsibility question, as well as different types of response mechanisms such as multi-item scales (see, however, Voiklis et al., 2016, and Stuart & Kneer, 2021 who report similar findings with different formulations and free-text responses). Moreover, moral responsibility and blame attributions can be susceptible to bias, which tends to be mitigated by within-subjects designs (see e.g. Spranca et al., 1991 and Kneer & Skoczeń, 2023). One could thus attempt to replicate our findings with a study in which participants asses moral responsibility in *both* the human and the AI pilot vignette. *Third*, further moderators of interest (context, agentic structure, severity of outcome, anthropomorphism etc.) must be investigated to get clearer on which factors influence our moral-psychological dispositions in HRI. *Fourth*: Given the important implications of retribution gaps, we should work towards a better understanding regarding the *mechanism* of human moral judgment in HRI. Most urgently, the question as to *why* we found a considerable willingness to hold autonomous systems morally responsible needs urgent attention. One possibility is that people misconceive the capacities of autonomous systems, and attribute inculpating mental states such as malicious intentions (Kneer, 2021) or recklessness to them (Kneer & Stuart, 2021; Stuart & Kneer, 2021). Another possibility is that the “intentional stance” (Dennett, 1987), a heuristic to save cognitive resources to make sense of the world, overshoots and we attribute blame though we do not really think that autonomous systems have intentions or foreknowledge (see Perez-Osorio & Wykowska, 2020; Marchesi et al., 2019; Schellen & Wykowska, 2019). *Fifth*, our results are characterized by a high degree of cross-cultural convergence (for similar convergence across the US and Japan concerning robot blame, see Komatsu et al., 2021). However, note that the three populations tested are quite similar in several respects. Although the cultures tested are at least not all WEIRD^10^ our samples nonetheless all belong to educated, industrialized, rich and democratic cultures (they are thus all “EIRD”). Future research should explore the important questions raised across a much larger number of cultures and languages, across which moral judgments have been shown to differ sometimes (see e.g. Barrett et al., 2016 and Stich & Machery, 2023, though compare Knobe, 2023). *Finally*, given that descriptive assumptions evidently matter for the debate concerning responsibility and retribution gaps, it stands to reason for practical philosophers to take findings from the emerging field of empirical HRI into account. In particular, when it comes to implications and policy recommendations, philosophers’ speculations might, by themselves, be too fragile a foundation to build on.

## Conclusion

According to the *Control Principle*, an agent is only morally responsible for outcomes that are sufficiently under their control. Certain philosophers, such as Matthias (2004) and Sparrow (2007), have questioned whether the required degree of control is present in certain types of human-AI interactions, and concluded that responsibility gaps can arise: Situations, that is, in which humans employ AI-driven systems, in which no human agent can justly be deemed at fault for harmful outcomes. Danaher (2016) has brought such normative, moral-philosophical considerations into dialogue with our descriptive, moral-psychological dispositions, which he considers strongly retributive. In human-AI interaction, he argues, retribution gaps can arise when the desire to blame *somebody* drives us to inappropriately attribute moral responsibility where none should be ascribed.

In our cross-cultural experiment, we have investigated retribution gaps empirically. With astonishing homogeneity across the three cultures explored (the US, Japan and Germany), we found that people are indeed willing to hold human agents responsible for harm occasioned by AI systems on their watch. Responsibility gap sceptics will welcome this result, in so far as our moral-psychological dispositions are consistent with their normative view. Advocates of responsibility gaps, by contrast, will be concerned, and – like Danaher – point to the potential dangers that can arise from this mismatch between ethics and our moral psychology. The latter range from inadvertent *misplacement* of blame via the purposeful *manipulation* of blame to the theoretical possibility of a *threat to the rule of law* more generally.

A second finding of ours, will be of concern to all parties to the debate, and relate directly to the three core worries: People manifest an astonishing disposition to hold AI systems themselves morally responsible, despite a broad-ranging philosophical consensus that such systems are not an appropriate target for moral blame. These results, which are consistent with some previous studies in moral HRI, deserve considerably more attention in future work, as their social implications could be very serious in the face of the increasing development and employment of artificial intelligence in high-stakes domains.

## Supplementary Information

Below is the link to the electronic supplementary material.

Supplementary file1 (PDF 185 KB)

## Footnotes

1 For interesting comparative analysis of different notions of “autonomous weapon systems”, see Taddeo & Blanchard (2022a) as well as Wood (2023a, 2023b).

2 Both Sparrow’s analogy of holding parents responsible and the lack of moral responsibility due to a lack of punishability have been objected to, see e.g. Leveringhaus (2016, 2018), for discussion see e.g. Santoni de Sio & Mecacci (2021).

3 List (2021) makes an interesting proposal, according to which AI systems could qualify as responsible agents similar to corporations. Kneer & Stuart (2021) have tested this proposal empirically and find that people do judge reckless AI systems akin to group agents.

4 For interesting discussion in this context, see Kraaijeveld (2020).

5 Two brief remarks regarding this apparent risk of retribution gaps: First, it is not quite true that “[p]sychological evidence suggests humans are innate retributivists” (2016, 299), as Danaher alleges, pointing to (somewhat dated) work by Carlsmith & Darley (2008) and Jensen (2010). The evidence is actually mixed and many psychologists, in particular Fiery Cushman, have produced a plethora of data in favour of pro-social accounts of punishment (for a review, see e.g. Cushman, 2015). Second, retributionism is principally a theory of *punishment*, and according to most ethicists its central considerations do not necessarily carry over neatly to debates regarding the nature and ethics of *blame* (see Coates & Tognazzini 2012 for an overview of positions). What is more, recent empirical work on folk morality suggests that the attribution of blame and punishment are sensitive to different factors and most likely driven by distinct cognitive processes (see Kneer & Machery, 2019, Kneer & Skoczen 2023, and Frisch et al., 2021, challenging Cushman 2008, who claims the contrary). If these findings were to replicate, we should be particularly careful not to conflate retributivism proper (regarding punishment) and a blame-related analogue. Given limitations of space, however, we will set these points aside for the purposes of this paper.

6 Pilot experiments with US participants showed that moral responsibility was consistently attributed to higher extents to human agents than to autonomous weapon systems. The effect sizes were close to large. An a priori power calculation with  α = .05, power = .80 and a mid-sized effect of *d* = .6 (one-tailed) suggested a required sample size of 72 participants, which we exceeded considerably in each country.

7 The complete materials and the data can be accessed via the project’s OSF page at https://osf.io/mcjg3/.

8 As a *post-hoc* power calculation conducted with G*Power 3.1 demonstrates, the probability of finding a medium-small effect (*d* = .50) with α = .05 – if there were one to be found – exceeds 99%. This also holds for the mean responsibility of the pilot-commander team reported below (see Fig. 4).

9 An anonymous reviewer suggests that people might attribute moral responsibility to robots in order to satisfy the expectations of the experimenters. This possibility (for any consciously chosen response) can never be ruled out completely in experimental work of the sort reported. Our findings are consistent with the attribution of moral responsibility and blame in a large number of other studies (see references at the end of Sect. "Retribution Gaps and their Implications"). Importantly, the findings from studies employing forced-choice and Likert scale response mechanisms concerning moral variables are consistent with findings from other experimental designs: In a study by Voiklis et al. (2016), for instance, participants were asked to provide free text responses to assess the actions of robots and humans, which were assessed with respect to a plethora of categories by several human coders. The results suggest that “people’s moral judgments of both agents relied on the same conceptual and justificatory foundation “, which – according to Voiklis et al. – means “that people extend moral cognition to nonhuman agents “ (Voiklis et al., 2016, p.1). In a more recent study by Stuart & Kneer (2021), participants were asked to rate both the moral qualities and potentially inculpating mental states (such as foreknowledge) of artificial agents. When given the possibility to downgrade inculpating mental states attributed to robots to metaphorical versions thereof (e.g. “knowledge”, in scare quotes and the like), participants stuck to knowledge proper – which also suggests that they see (certain types of) artificial agents as more human-like than one might suppose.

10 WEIRD stands for Western, Educated, Intentional, Rich, and Democratic cultures. For a manifesto that behavioral science has to go beyond the near-exclusive sampling of US Americans and WEIRD people more general, see e.g. Henrich et al. (2010) and Henrich (2020).
