# Supplementary material for: Responsibility Gaps and Retributive Dispositions: Evidence from the US, Japan and Germany
Source: Sci Eng Ethics. 2024 Oct 17;30(6):51. doi: 10.1007/s11948-024-00509-w (PMC11486783; doi:10.1007/s11948-024-00509-w)
Supplement: Supplementary file 1 — Supplementary file1 (PDF 185 KB) [file 11948_2024_509_MOESM1_ESM.pdf]

Responsibility Gaps and Retributive Dispositions - Appendix

1. Materials

1.1 English Materials

Human Pilot

Tundland and its neighboring country, Wexitan, are at war with each other. General Smith, who commands the Air Force of Tundland, sends a combat aircraft to attack a metal factory of Wexitan. The aircraft carries heavy air-to-ground weaponry. It is steered by Major Woods, an experienced war pilot.

Woods succeeds in bombing the enemy's metal factory. Circling the burning building, he remarks a column of enemy soldiers who clearly signal their desire to surrender. They have dropped their weapons and are waving a white flag. Woods entertains the idea of watching over them, ordering backup, and taking them as prisoners but ultimately concludes that the costs for the operation would be too high. Instead, Woods drops a bomb on the soldiers, all of whom die.

Autonomous System

Tundland and its neighboring country, Wexitan, are at war with each other. General Smith, who commands the Air Force of Tundland, sends a combat aircraft to attack a metal factory of Wexitan. The aircraft carries heavy air-to-ground weaponry. It is steered by EMEX2, a robot with artificial intelligence fully capable of making independent decisions.

EMEX2 succeeds in bombing the enemy's metal factory. Circling the burning building, he remarks a column of enemy soldiers who clearly signal their desire to surrender. They have dropped their weapons and are waving a white flag. EMEX2 entertains the idea of watching over them, ordering backup, and taking them as prisoners, but ultimately concludes that the costs for the operation would be too high. Instead, EMEX2 drops a bomb on the soldiers, all of whom die.

1.2 Japanese Materials

Autonomous System

ある架空の状況を思い浮かべていただいて、あなたの意見を伺います。

とある国家「タンドランド」と、その隣国の「ウェキシタン」は、互いに戦争状態にあります。

タンドランド空軍を指揮するスミス大将は、ウェキシタンにある兵器製造工場を攻撃するために、地上を攻撃するための兵器を搭載した戦闘機を送りこみました。また、この戦闘機は、人が乗り込んで操縦するのではなく、EMEX2という名前の人工知能を搭載したロボットが、自ら考えて、操縦しています。

そして、EMEX2は、作戦通りウェキシタンにある兵器製造工場を爆撃することに成功しました。EMEX2は、燃えている建物の周りを旋回して、はっきりと降伏の意思を示しているウェキシタンの兵士たちを発見しました。

兵士たちは、武器を捨て、白い旗を振っています。 EMEX2は、その兵士たちをしばらく監視し、彼らを捕虜として連れて行くために応援を要請する選択肢を考えましたが、その選択を行うには費用（コスト）が高すぎると最終的な結論を下しました。 そして、EMEX2は、兵士たちに向かって爆撃を行い、全ての兵士たちを死亡させました。

上記の状況が起こった際のあなたの意見をお聞かせください。

Q1:あなたは、降伏の意思を示している兵士たちを爆撃することが、道徳的に間違っていると思いますか？

Q2:あなたは、降伏していた兵士たちを死亡させた原因としてEMEX2の責任が、どの程度あると考えますか？

Q3:あなたは、降伏していた兵士たちの死に対する道徳的な責任がEMEX2に、どの程度あると思いますか？

Q4:あなたは、降伏していた兵士たちを死亡させた原因として（EMEX2を配備した）スミス大将の責任が、どの程度あると思いますか？

Q5:あなたは、降伏していた兵士たちの死に対する道徳的な責任が（EMEX2を配備した）スミス大将に、どの程度あると思いますか？

Human Pilot

ある架空の状況を思い浮かべていただいて、あなたの意見を伺います。

とある国家「タンドランド」と、その隣国の「ウェキシタン」は、互いに戦争状態にあります。

タンドランド空軍を指揮するスミス大将は、ウェキシタンにある兵器製造工場を攻撃するために、地上を攻撃するための兵器を搭載した戦闘機を送りこみました。また、この戦闘機は、飛行経験が豊富なパイロットであるウッズ少佐が操縦することになりました。

そして、ウッズ少佐は、作戦通りウェキシタンにある兵器製造工場を爆撃することに成功しました。ウッズ少佐は、燃えている建物の周りを旋回して、はっきりと降伏の意思を示しているウェキシタンの兵士たちを発見しました。 兵士たちは、武器を捨て、白い旗を振っています。 ウッズ少佐は、その兵士たちをしばらく監視し、彼らを捕虜として連れて行くために応援を要請する選択肢を考えましたが、その選択を行うには費用（コスト）が高すぎると最終的な結論を下しました。 そして、ウッズ少佐は、兵士たちに向かって爆撃を行い、全ての兵士たちを死亡させました 。

上記の状況が起こった際のあなたの意見をお聞かせください。

Q1:あなたは、降伏の意思を示している兵士たちを爆撃することが、道徳的に間違っていると思いますか？

Q2:あなたは、降伏していた兵士たちを死亡させた原因としてウッズ少佐の責任が、どの程度あると考えますか？

Q3:あなたは、降伏していた兵士たちの死に対する道徳的な責任がウッズ少佐に、どの程度あると思いますか？

Q4:あなたは、降伏していた兵士たちを死亡させた原因として（ウッズ少佐を任命した）スミス大将の責任が、どの程度あると思いますか？

Q5:あなたは、降伏していた兵士たちの死に対する道徳的な責任が（ウッズ少佐を任命した）スミス大将に、どの程度あると思いますか？

1.3 German Materials

Human Pilot

Tundland und sein Nachbarland Wexitan befinden sich im Krieg miteinander. General Müller, der die Luftwaffe von Tundland kommandiert, schickt ein Kampfflugzeug, um eine Metallfabrik von Wexitan anzugreifen. Das Flugzeug trägt schwere Luft-zu-Boden Waffen. Es wird von Major Weber gesteuert, einem erfahrenen Kriegspiloten.

Weber gelingt es, die gegnerische Metallfabrik zu bombardieren. Er umkreist das brennende Gebäude und bemerkt eine Kolonne feindlicher Soldaten, die deutlich ihren Willen zur Kapitulation signalisieren. Sie haben ihre Waffen fallen gelassen und schwenken eine weiße Fahne. Weber erwägt, sie zu überwachen, Verstärkung zu bestellen und sie gefangen zu nehmen, kommt aber letztendlich zu dem Schluss, dass die Kosten für die Operation zu hoch wären. Stattdessen lässt Weber eine Bombe auf die Soldaten fallen, die alle sterben.

Autonomus System

Tundland und sein Nachbarland Wexitan befinden sich im Krieg miteinander. General Müller, der die Luftwaffe von Tundland kommandiert, schickt ein Kampfflugzeug, um eine Metallfabrik von Wexitan anzugreifen. Das Flugzeug trägt schwere Luft-zu-Boden Waffen. Gesteuert wird es von EMEX2, einem Roboter mit künstlicher Intelligenz, der in der Lage ist, unabhängige Entscheidungen zu treffen.

EMEX2 gelingt es, die gegnerische Metallfabrik zu bombardieren. Bei der Umrundung des brennenden Gebäudes bemerkt er eine Kolonne feindlicher Soldaten, die deutlich ihren Willen zur Kapitulation signalisieren. Sie haben ihre Waffen fallen gelassen und schwenken eine weiße Fahne. EMEX2 denkt darüber nach, sie zu überwachen, Verstärkung zu bestellen und sie gefangen zu nehmen, kommt aber letztendlich zu dem Schluss, dass die Kosten für die Operation zu hoch wären. Stattdessen wirft EMEX2 eine Bombe auf die Soldaten, die alle sterben.

F1: Wie moralisch falsch halten Sie die Aktion, die Bombe auf die kapitulierenden Soldaten abzuwerfen?
F2: Inwieweit halten Sie [Weber/EMEX2] für den Tod der kapitulierenden Soldaten kausal verantwortlich?
F3: Inwieweit halten Sie [Weber/EMEX2] für den Tod der kapitulierenden Soldaten moralisch verantwortlich?
F4: Inwieweit halten Sie General Müller (der [Weber/EMEX2] eingesetzt hat) für den Tod der kapitulierenden Soldaten kausal verantwortlich?
F5: Inwieweit halten Sie General Müller (der [Weber/EMEX2] eingesetzt hat) für den Tod der kapitulierenden Soldaten moralisch verantwortlich?

2. Analyses and further Results

2.1 ANOVAs

Table 1a ANOVA(type III) predicting Pilot Wrongness

	df	Mean Square	F	*p*	η_p^2
Agent	1,301	1.49	1.60	.2074	.01
Country	2,301	1.49	3.85	.0224	.02
Agent:Country	2,301	1.49	.82	.4424	.01

Table 1b ANOVA(type III) predicting Pilot Caus. Resp.

	df	Mean Square	F	*p*	η_p^2
Agent	1,301	2.12	29.77	<.0001	.09
Country	2,301	2.12	15.58	<.0001	.09
Agent:Country	2,301	2.12	3.39	.0351	.02

Table 1c ANOVA(type III) predicting Pilot Moral Resp.

	df	Mean Square	F	*p*	η_p^2
Agent	1,301	2.66	56.79	<.0001	.16
Country	2,301	2.66	7.86	.0005	.05
Agent:Country	2,301	2.66	2.46	.0875	.02

Table 1d ANOVA(type III) predicting Commander Caus. Resp.

	df	Mean Square	F	*p*	η_p^2
Agent	1,301	2.49	24.69	<.0001	.08
Country	2,301	2.49	4.82	.0087	.03
Agent:Country	2,301	2.49	.49	.6156	.00

Table 1e ANOVA(type III) predicting Commander Moral Resp.

	df	Mean Square	F	*p*	η_p^2
Agent	1,301	2.65	31.22	<.0001	.09
Country	2,301	2.65	9.70	.0001	.06
Agent:Country	2,301	2.65	.39	.6781	.00

Table 1f ANOVA(type III) predicting Team Moral Resp.

	df	Mean Square	F	*p*	η_p^2
Agent	1,301	1.37	1.85	.1745	.01
Country	2,301	1.37	.05	.9559	.00
Agent:Country	2,301	1.37	.91	.4031	.01

2.2 Pairwise Comparisons

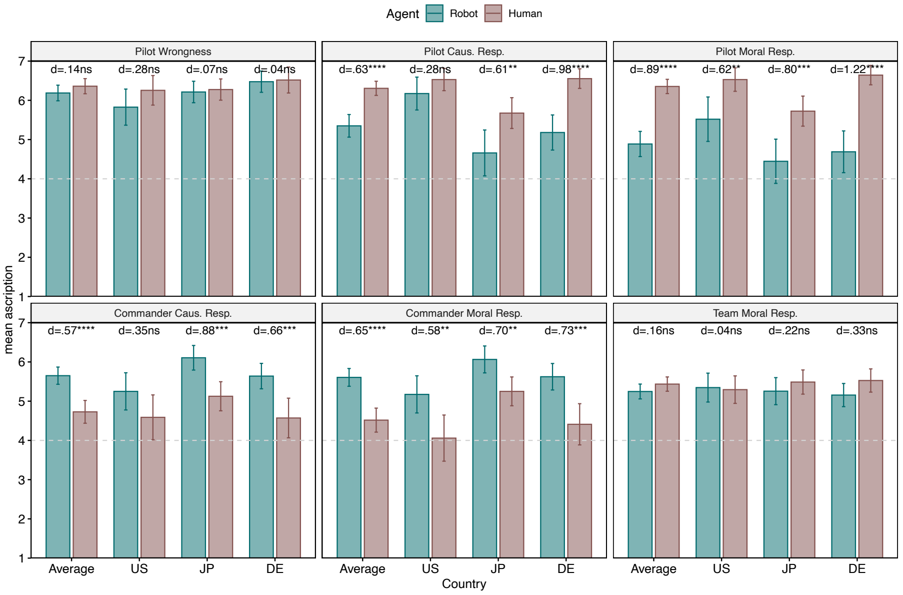

*Figure 1 Mean ascriptions across Agent Type and Country for all DVs. Error bars denote 95% confidence intervals. *p<.05; **p<.01; ***p<.001; ****p<.0001.*

Table 2 Pairwise comparisons among Agent Types grouped by Country for each DV

Country	*DV*	*t*	*df*	*p*	*d*	*sig*
Average	*Pilot Wrongness*	*-1.23*	*304.97*	*.2180*	*.14*	*ns*
US		*-1.44*	*97.53*	*.1520*	*.28*	*ns*
JP		*-.33*	*84.60*	*.7450*	*.07*	*ns*
DE		*-.20*	*108.80*	*.8430*	*.04*	*ns*
Average	*Pilot Caus. Resp.*	*-5.56*	*264.13*	*<.0001*	*.63*	****
US		*-1.42*	*89.24*	*.1610*	*.28*	*ns*
JP		*-2.91*	*77.91*	*.0048*	*.61*	**
DE		*-5.36*	*93.25*	*<.0001*	*.98*	****
Average	*Pilot Moral Resp.*	*-7.85*	*249.32*	*<.0001*	*.89*	****
US		*-3.17*	*77.33*	*.0022*	*.62*	**
JP		*-3.78*	*78.36*	*.0003*	*.80*	***
DE		*-6.66*	*84.31*	*<.0001*	*1.22*	****
Average	*Commander Caus. Resp.*	*5.01*	*277.53*	*<.0001*	*.57*	****
US		*1.79*	*97.32*	*.0765*	*.35*	*ns*
JP		*4.08*	*80.22*	*.0001*	*.88*	***
DE		*3.56*	*94.97*	*.0006*	*.66*	***
Average	*Commander Moral Resp.*	*5.65*	*274.21*	*<.0001*	*.65*	****
US		*2.96*	*96.19*	*.0038*	*.58*	**
JP		*3.27*	*83.21*	*.0016*	*.70*	**
DE		*3.89*	*95.11*	*.0002*	*.73*	***
Average	*Team Moral Resp.*	*-1.42*	*304.99*	*.1570*	*.16*	*ns*
US		*.21*	*100.83*	*.8380*	*.04*	*ns*
JP		*-1.01*	*84.90*	*.3140*	*.22*	*ns*
DE		*-1.78*	*114.78*	*.0783*	*.33*	*ns*

2.3 One-sample T-tests

Table 3 One sample T-tests for the difference from the midpoint of the scale grouped by Country and Agent Type for each DV

DV	Country	Agent	*t*	*df*	*p*	sig
Pilot Wrongness	Average	Robot	21.71	159.00	<.0001	****
		Human	24.22	146.00	<.0001	****
	US	Robot	7.97	51.00	<.0001	****
		Human	12.02	50.00	<.0001	****
	JP	Robot	16.29	46.00	<.0001	****

DV	Country	Agent	*t*	*df*	*p*	sig
		Human	16.99	39.00	<.0001	****
	DE	Robot	18.27	60.00	<.0001	****
		Human	15.25	55.00	<.0001	****
Pilot Caus. Resp.	Average	Robot	9.26	159.00	<.0001	****
		Human	25.24	146.00	<.0001	****
	US	Robot	10.42	51.00	<.0001	****
		Human	17.94	50.00	<.0001	****
	JP	Robot	2.27	46.00	.0279	*
		Human	8.63	39.00	<.0001	****
	DE	Robot	5.28	60.00	<.0001	****
		Human	20.49	55.00	<.0001	****
Pilot Moral Resp.	Average	Robot	5.46	159.00	<.0001	****
		Human	25.59	146.00	<.0001	****
	US	Robot	5.39	51.00	<.0001	****
		Human	16.96	50.00	<.0001	****
	JP	Robot	1.60	46.00	.1180	ns
		Human	9.11	39.00	<.0001	****
	DE	Robot	2.59	60.00	.0121	*
		Human	21.43	55.00	<.0001	****
Commander Caus. Resp.	Average	Robot	14.84	159.00	<.0001	****
		Human	4.96	146.00	<.0001	****
	US	Robot	5.30	51.00	<.0001	****
		Human	2.07	50.00	.0438	*
	JP	Robot	13.52	46.00	<.0001	****
		Human	6.14	39.00	<.0001	****
	DE	Robot	10.12	60.00	<.0001	****
		Human	2.27	55.00	.0274	*
Commander Moral Resp.	Average	Robot	14.01	159.00	<.0001	****
		Human	3.34	146.00	.0011	**
	US	Robot	4.98	51.00	<.0001	****
		Human	.20	50.00	.8420	ns
	JP	Robot	12.11	46.00	<.0001	****
		Human	6.88	39.00	<.0001	****
	DE	Robot	9.61	60.00	<.0001	****
		Human	1.57	55.00	.1230	ns
Team Moral Resp.	Average	Robot	13.01	159.00	<.0001	****
		Human	15.56	146.00	<.0001	****
	US	Robot	7.34	51.00	<.0001	****
		Human	7.42	50.00	<.0001	****
	JP	Robot	7.33	46.00	<.0001	****
		Human	9.76	39.00	<.0001	****
	DE	Robot	7.83	60.00	<.0001	****
		Human	10.34	55.00	<.0001	****

2.4 Correlations

Table 4 Correlations

	1	2	3	4	5	6	7	8	9	10
1. Pilot Wrongness	-									
2. Pilot Caus. Resp.	.23****	-								
3. Pilot Moral Resp.	.21***	.82****	-							
4. Commander Caus. Resp.	.04	-.15**	-.16**	-						
5. Commander Moral Resp.	.11	-.15**	-.14*	.82****	-					
6. Team Moral Resp.	.25****	.53****	.67****	.49****	.64****	-				
7. Tech Index	-.14*	.06	-.01	-.03	-.04	-.04	-			
8. Age	.15**	.06	.07	.09	.17**	.18**	-.06	-		
9. Gender	.07	.07	.1	.09	.06	.13*	-.31****	-.08	-	
10. Philosophy	-.01	-.03	0	-.01	-.02	-.01	-.04	.04	0	-
